# Tubulin Post-translational Modifications: Potential Therapeutic Approaches to Heart Failure

**DOI:** 10.3389/fcell.2022.872058

**Published:** 2022-04-12

**Authors:** Chang Liu, Yuwen Chen, Yao Xie, Meixiang Xiang

**Affiliations:** Department of Cardiology, The Second Affiliated Hospital, Zhejiang University School of Medicine, Hangzhou, China

**Keywords:** tubulin post-translational modification, microtubule, heart failure, cardiovascular disease, detyrosination, acetylation, glutamylation, glycylation

## Abstract

In recent decades, advancing insights into the mechanisms of cardiac dysfunction have focused on the involvement of microtubule network. A variety of tubulin post-translational modifications have been discovered to fine-tune the microtubules’ properties and functions. Given the limits of therapies based on conserved structures of the skeleton, targeting tubulin modifications appears to be a potentially promising therapeutic strategy. Here we review the current understanding of tubulin post-translational modifications in regulating microtubule functions in the cardiac system. We also discussed how altered modifications may lead to a range of cardiac dysfunctions, many of which are linked to heart failure.

## Introduction

Microtubules, polymers of αβ-tubulin, are major cytoskeletal components in all eukaryotic cells and are involved in multiple cellular events, including cell shape, polarization, intracellular trafficking, mitosis, axonemal-based motility and morphogenesis (Nogales, 2001). In the cardiovascular system, the microtubule network actively participates in heart development, cardiac excitability and contractility, cargo molecule assembly and molecular trafficking, and cilia-related functions (Cooper, 2006; Nishimura et al., 2006; Pala et al., 2018; Caporizzo et al., 2019; Djenoune et al., 2021). Cardiac microtubule networks act as mechanotransducers (Prosser et al., 2011) and structural compression-resistant elements that buckle against the load upon contraction (Granzier and Irving, 1995; Nishimura et al., 2006). Deregulations of these mechanical characteristics have been proved to be correlated with pathological conditions such as heart failure and cardiac hypertrophy where the heart is subjected to persistent high loads (White, 2011). Further, cardiovascular cilia abnormalities are linked to congenital heart failure (Pala et al., 2018), and vascular abnormal fluid shear stress sensing diseases including hypertension, aneurysms, and atherosclerosis (Pala et al., 2018; Wang et al., 2021). Although the microtubule structure is conserved across cell types in different organisms, they may be adapted to a wide range of functionally specialized tasks. The intrinsic “tubulin code”, which includes subunit isoforms and post-translational modifications (PTMs), is responsible for the filament’s adaptation to various specific functions due to the filament’s extreme conservation of *a*- and *ß*-tubulins (Janke and Magiera, 2020).

In recent years, advancing insights into the mechanisms of cardiovascular dysfunction have focused on the role of the microtubule network. Microtubule-targeting drugs, which interfere with microtubule dynamics, are one of the most successful first-line cancer therapies (Wordeman and Vicente, 2021). However, systemic administration provokes a range of negative side effects on other systems (Miltenburg and Boogerd, 2014). In the light of limitations of treatments on the overall microtubule (Stamp, 2014), researchers have turned their interest to the study of PTM which would have higher selectivity. Additionally, evidence of the PTM’s role in the cardiovascular system has accumulated, especially in the function of myocardial microtubules and the MT-based organelle, primary cilia. Therefore, targeting the identified role of the tubulin PTM tends to be a potentially viable therapeutic option. In the review, we summarized the roles of the tubulin modifications—detyrosination/re-tyrosination, acetylation, glutamylation and glycosylation in regulation of the microtubule function, mainly focusing on the cardiomyocytes and primary cilium. Furthermore, potential links between PTMs and cardiac dysfunction that may progress to heart failure are discussed.

## The Microtubule Network and PTMs in the Cardiac System

**FIGURE 1:**
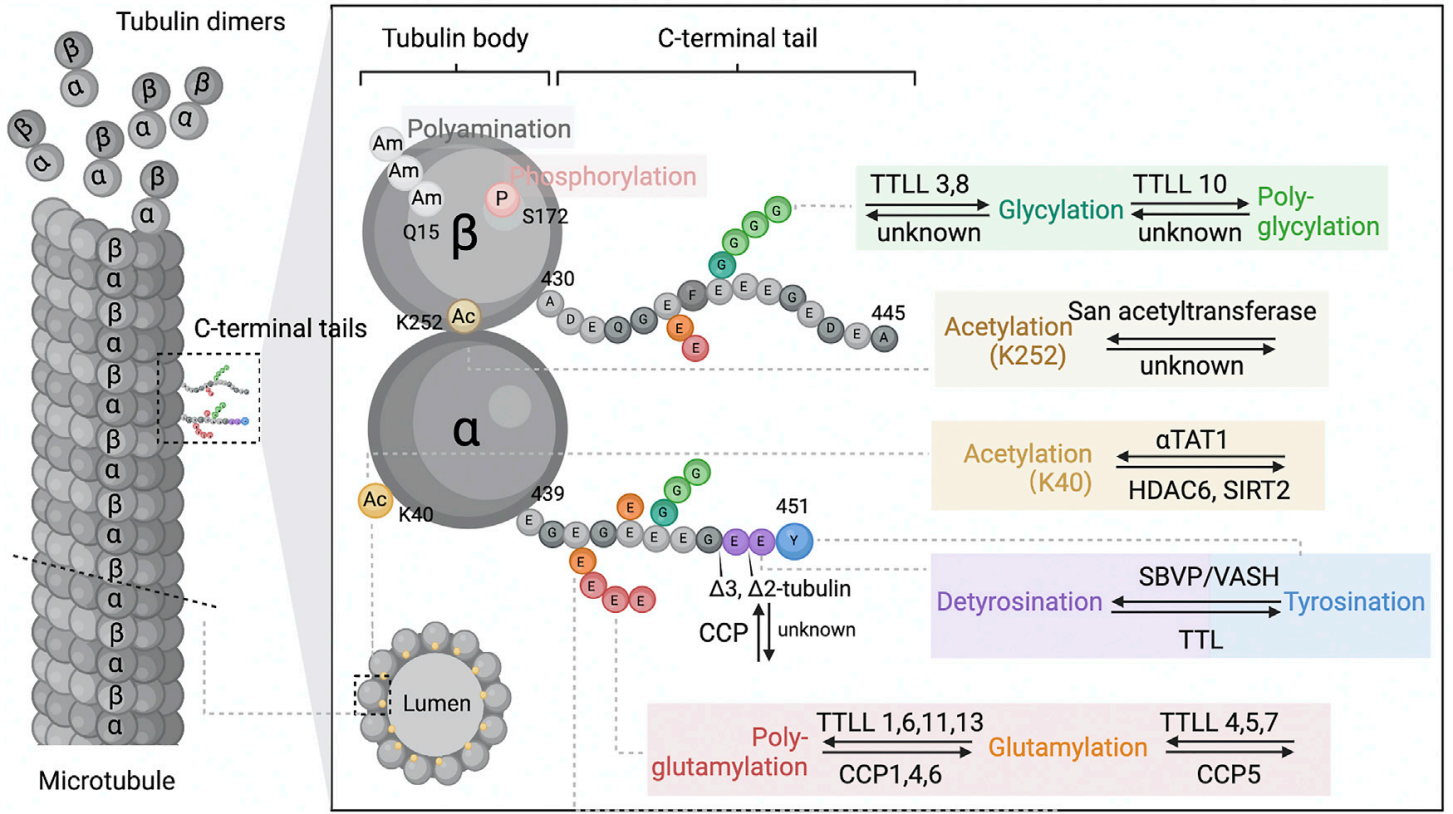
Microtubule and tubulin post-translational modifications. Microtubules dynamically assemble from head-to-tail arrays of αβ-tubulin dimers. The tubulin is globular and highly conserved protein that forms a “tubulin body”. The tubulin C-terminal amino acid tails are unstructured segments protruding from the surface of the ordered tubulin body and may regulate microtubule intrinsic properties and the binding behaviour of microtubule associated proteins. Tubulin post-translational modifications occur either at specified sites of the tubulin body (Ac, acetylation; P, phosphorylation; Am, polyamination), or within the C-terminal tails (detyrosination and detyrosination, glutamylation, glycylation, ∆2-tubulin and ∆3-tubulin prodeced by glutamate residue removals). These modifications are often dynamic reversible and catalysed by a range of enzymes from multiple families. During the process, the functional properties of microtubules are changed by single residue alternations (acetylation, phosphorylation, detyrosination) or modulating the non-binary signals by elongation of the side chains (polyamination, polyglutamylation, polyglycylation). Abbreviations: TTLL, tubulin–tyrosine ligase-like family; αTAT1, *a*-tubulin N-acetyltransferase 1; HDAC, tubulin-lysine deacetylase; SIRT, sirtuin; SVBP, vasohibin binding protein; VASHs, vasohibins; TTL, tubulin-tyrosine ligase-like family; CCP, cytosolic carboxypeptidase-like protein; K40, lysine 40.

The building blocks of a microtubule structure are heterodimeric αβ-tubulin heterodimers, which are arranged head-to-tail into polar protofilaments with *a*- and *ß*-tubulin exposed at either end. Ten to fifteen linear protofilaments (usually thirteen in mammalian cells) associate laterally to form a hollow microtubule cylinder (Figure 1) (Tilney et al., 1973). Many of the microtubules’ functions are accomplished by their assembly into specialized intracellular structures, such as the mitotic spindle or the cilia/flagella axoneme (Gadadhar et al., 2017a). Microtubules intrinsically undergo dynamic instability, cycling between growth and depolymerization (Mitchison and Kirschner, 1984). In addition, flexural rigidity is an intrinsic mechanical property of microtubules (Gittes et al., 1993) required for a variety of cellular activities. As the stiffest cytoskeletal components, mechanical bending requires neighboring protofilaments to slide against one another by overcoming interprotofilament contact resistance (Mehrbod and Mofrad, 2011). The loss of this interaction can reduce the bending rigidity of microtubules, making microtubules more resistant to mechanical breakage or disassembly (Xu et al., 2017).

**FIGURE 2:**
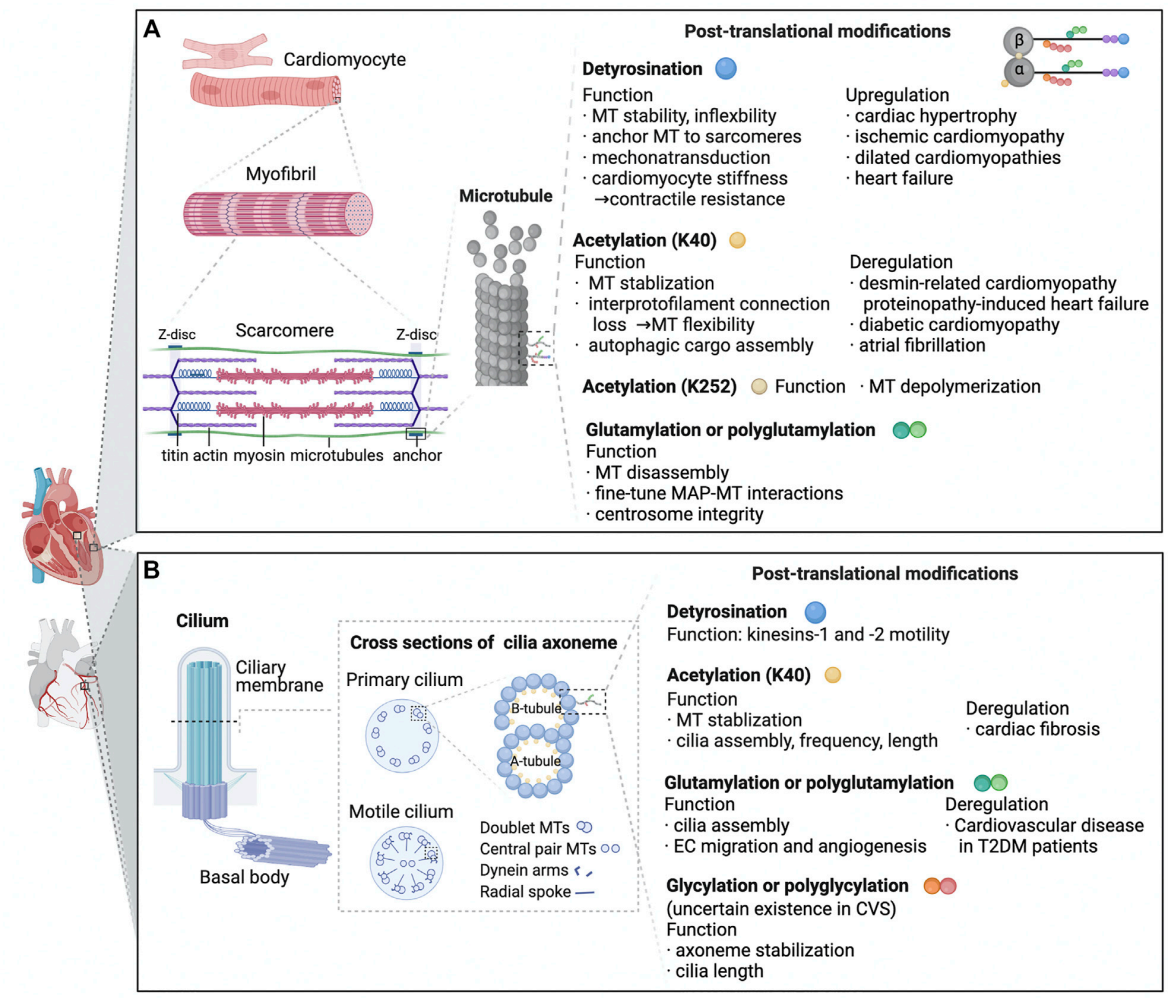
Known functions and pathologic roles of tubulin post-translational modifications (PTMs) in cardiomyocytes and cardiac primary cilia are depicted. **(A)** Myocardiocytes. In cardiomyocytes, microtubules predominantly grow along the long axis of the cell, interdigitating with the myofibrils. In a beating heart, when sarcomeres shorten and stretch, microtubules buckle under load. PTMs generated on the tubulin body and C-terminal tails possess various functions which are essential for microtubule properties, MAP interactions and cell contraction. As listed in the figure, deregulation of PTMs is linked to a range of cardiac dysfunctions. **(B)** Cilia. A cilium assembles from the axoneme contiguous with the basal body. The axoneme comprises doublets built by 13 tubulin protofilaments (A-tubule), and an incomplete microtubule containing 10 protofilaments (B-tubule). In 9 + 2 motile cilia, the 9 doublets are integrated into a cylindric array with radial spokes and dynein arms. Primary 9 + 0 cilia do not possess dynein arms, radial spokes or central pairs. Axonemal microtubules are modified by a range of tubulin PTMs, exerting various effects on microtubules and cilia-related disorders. Abbreviations: K40, lysine 40; MT,microtubule; K252, lysine 252; MAP, microtubule-associated protein; EC, endothelial cells; T2DM, type 2 diabetes mellitus; CVS, cardiovascular system.

Microtubules, along with intermediate filaments and actin, constitute the integral components of the cardiomyocyte cytoskeleton (Figure 2). In cardiac myocytes, the microtubule network is oriented towards the nucleus and aligns longitudinally along the myofibrillar matrix (Eckel and Reinauer, 1983), where it is thought to function as a dynamic transport mechanism around mitochondria and along the plasma membrane (Eckel and Reinauer, 1983). Together with intermediate filaments, the network is involved in myofilament initial assembly and adult maintenance (Hein et al., 2000; Kostin et al., 2000; Sequeira et al., 2014; Childers et al., 2019). Microtubules structures around the nucleus and primarily organized in the longitudinal myofibril space, where they are originally suggested to act as a dynamic transportation system (Sequeira et al., 2014). In the past decade, microtubules have garnered considerable interest due to their involvement in the beating heart and in maintaining cardiomyocyte health. Except for its well-defined transportation function for vesicles and mRNA, the cardiac microtubule network possesses multiple mechanical roles in the beating myocyte and in pathological conditions such as heart failure and cardiac hypertrophy when the heart is subjected to persistent high loads (White, 2011). Microtubules work as mechanotransducers, transforming contractile forces into intracellular signals (Prosser et al., 2011). They may also act as structural compression-resistant elements that buckle and bear the load upon cardiomyocyte contraction (Granzier and Irving, 1995; Nishimura et al., 2006).

Since increasing evidence has revealed the critical roles of defective cilia in the pathogenesis of cardiovascular diseases, the function of cardiac cilia has become a growing concern in recent years. Cilia are microtubule-based organelles extending from the apical surfaces of most animal cells. It is made up of an axoneme with nine circumferentially aligned doublet microtubules that stretch outwards from the centriole/basal body into the extracellular matrix (Figure 2) (Satir and Christensen, 2007). Traditionally, cilia are categorized into motile and primary cilia (also known as non-motile cilia) (Satir and Christensen, 2007). Both primary and motile cilia are required for the cardiac left–right asymmetry patterning during heart development (Djenoune et al., 2021), while primary cilia have been recognized as having essential functional roles in the embryonic, neonatal and adult hearts. Primary cilia are found mostly in quiescent cells (Berbari et al., 2009) and participate in a variety of cellular functions, for example, cell proliferation, differentiation, cycle regulation and mechanochemical sensing (Pampliega et al., 2013; Yuan et al., 2016; Villalobos et al., 2019). It is also a sensory organelle that senses mechanochemical signals on the apical cell membrane. Although several studies have characterized the presence of primary cilia in embryonic and adult heart (Rash et al., 1969; Myklebust et al., 1977; Diguet et al., 2015) and aortic endothelial cells (Bystrevskaya et al., 1992), the specific cell location and function of cilia in the cardiac system remain controversial. Sarbjot et al. recently described ciliated cells protruding from fibroblasts in a non-exclusive manner (Villalobos et al., 2019).

The primary cilium serves as a critical transduction center throughout heart development, orchestrating numerous signaling pathways involved in left-right asymmetry formation, haemodynamic mechanosensation, valvulogenesis, and myocardial regeneration (for a recent overview, see Djenoune et al., 2021). (Djenoune et al., 2021). Cilia structural or functional abnormalities are significantly linked to congenital heart defects, such as atrial and ventricular septal defects, abnormal cardiac looping and heart tube remodeling (Pala et al., 2018). These defects can result in congestive heart failure. Additionally, mutations in cilia structural genes are linked to severe heart phenotypes (Pala et al., 2018). In contrast, there is less information about the roles of cilia in adult heart function. Primary cilia are abundant in shear stress disturbed zones of the adult cardiovascular system, where they function as mechanosensors, transmitting extracellular stimuli into intracellular space (Wang et al., 2021). Furthermore, primary cilia located in the heart cavity protect endothelial cells from epithelial-mesenchymal transition, calcification, and flow-related damage. Primary cilia dysfunction leads to abnormal fluid shear stress sensing and, as a result, vascular diseases such as hypertension, aneurysms, and atherosclerosis (Pala et al., 2018; Wang et al., 2021).

Notably, since microtubules do not work alone, they are usually functionally specialized by interactions with a wide array of microtubule-associated proteins (MAPs). MAPs bind to microtubules to generate various effects, such as regulation of microtubule dynamics and stability, generating forces (motor proteins), connecting microtubules to other cellular components, or carrying cargo along microtubules (Bodakuntla et al., 2019). Furthermore, microtubules’ properties can also be programmed by tubulin isotypes and conserved PTMs (Gadadhar et al., 2017a). Among tubulin PTMs, tyrosination and detyrosination, acetylation, and polyglutamylation are the most studied (Janke and Magiera, 2020). Isolated microtubules would exhibit negligible minimal resistance to myocyte compression, whereas these mechanical behaviors can be changed by orders of magnitude through diverse PTM (Mehrbod and Mofrad, 2011). In terms of ciliary dynamics and function, tubulin PTMs are evidenced to influence cilia assembly, stability and motility.

## Tubulin Post-translational Modifications

### Detyrosination and Tyrosination

Detyrosination/re-tyrosination, first identified in 1973, is the most well-characterized reversible PTM (Barra et al., 1973). Detyrosination commences the detyrosination–tyrosination cycle by cleaving the gene-encoded C-terminal tyrosine residue at *a*-tubulin tail, which is then re-inserted during tyrosination (Gadadhar et al., 2017a). Tyrosination is catalyzed enzymatically by tubulin tyrosine ligase (TTL), identified nearly 30 years ago (Ersfeld et al., 1993) (Rogowski et al., 2009), but the enzyme responsible for detyrosination, vasohibins (VASHs) and vasohibin binding protein (SVBP) complex, was discovered very recently (Nieuwenhuis et al., 2017). Furthermore, due to the particular structural match between the TTL and tubulin, tyrosination has been proven to be specific to *a*-tubulin (Prota et al., 2013). In the heart, detyrosinated *a*-tubulin was discovered to be the most prevalent post-translationally modified tubulin, which helps to anchor microtubules to sarcomeres and regulate microtubule buckling during contraction (Belmadani et al., 2004; Kerr et al., 2015; Robison et al., 2016). For the cilium axoneme, detyrosination is abundant on the *ß*-tubule of outer doublet microtubule (Wloga et al., 2017) and associated with kinesins-1 and -2 motility (Kaul et al., 2014; Sirajuddin et al., 2014), but so far little is known about their involvement in the cardiovascular system.

Detyrosination has commonly been correlated with increased microtubule dynamic stability (Schulze et al., 1987) by preventing kinesin-13-mediated active depolymerization (Peris et al., 2009) and indirectly lowering microtubule development *via* cytoplasmic linker protein 170 (CLIP170) or dynactin subunit 1 (DCTN1) (Peris et al., 2006; Janke and Magiera, 2020). Given the association between microtubule dynamic instability and dysfunctional cardiovascular events such as cardiac hypertrophy, myocardial ischemia–reperfusion damage and heart failure (Sato et al., 1993; Tagawa et al., 1996), detyrosination may be critical in protecting cardiac long-lived microtubules from depolymerization.

Detyrosination modifications may potentially influence microtubule mechanics. Although tyrosine-dependent regulation of microtubule flexibility has yet to be proven, its involvement during striated muscle contraction suggests that tyrosine may act as a regulator of microtubule flexibility (Kerr et al., 2015). Increased detyrosination may enhance myocyte stiffness and impede contraction while offering little energy return during relaxation (Robison et al., 2016). Consistent with this, increased levels of detyrosination in human hearts have been related to clinical contractile malfunction (Robison et al., 2016). Still, it remains unknown whether detyrosination directly decreases the flexibility of microtubules or attracts associated-proteins to them, resulting in the alteration of mechanical properties. In the heart, the detyrosination of the microtubule network is emerging as a developing regulator for cardiac muscle mechanical performance (Kerr et al., 2015; Robison et al., 2016). Recent research indicates that detyrosination of tubulin affects microtubule-based cardiac mechanotransduction *via* ROS and Ca2+ signals (Kerr et al., 2015). By providing resistance to sarcomere shortening (Kerr et al., 2015), this change modulates cytoskeletal stiffness and alters muscular contractility (Kerr et al., 2015). In particular, tyrosinated regions of the network move easier with the myocyte, hence providing minimum resistance to contraction. Detyrosinated portions, in contrast, are believed to facilitate microtubule attachment to sarcomeres by establishing orthogonal grid complexes with desmin (intermediate filament), which then offer robust resistance to cardiomyocyte contraction when buckling under load. Strikingly, inhibited microtubule detyrosination leads to a sliding rather than buckling action of the microtubule, which increases the shortening and contractile velocity during systolic contraction (Robison et al., 2016). This has far-reaching consequences for microtubule load–bearing capacity, as well as altered mechanical resistance and mechano-signaling in heart failure (Yingxian Chen et al., 2018).

Cardiac muscle mechanical abnormalities have been correlated with upregulated tubulin detyrosination in a variety of heart diseases, such as ischemic cardiomyopathy, hypertrophic cardiomyopathies, myocardial infarction, dilated cardiomyopathies and heart failure (Belmadani et al., 2004; Yingxian Chen et al., 2018; Schuldt et al., 2021; Yu et al., 2021). In failing cardiomyocytes, microtubule networks were dense and highly detyrosinated, resulting in enhanced cardiomyocyte stiffness and decreased contractility, regardless of the disease cause (Yingxian Chen et al., 2018). In these findings, pharmacological or genetical suppression of microtubule detyrosination inhibits pathogenic development by blunting stress-induced ROS and Ca2+ signaling abnormalities (Kerr et al., 2015) and can recover 40–50% of lost contractile performance (Yingxian Chen et al., 2018). The inhibition of detyrosination improved relaxation dynamics in diastolic impaired cardiomyocytes (Yingxian Chen et al., 2018).

Notably, microtubule-affinity regulating kinase 4 (MARK4)-dependent modulation was recently discovered to be a mechanism for microtubule detyrosination during myocardial infarction (Yu et al., 2021). In cardiomyocytes, microtubule detyrosination could be finely tuned *via* the microtubule-associated protein 4 (MAP4) phosphorylation mechanism, which allows vasohibin’s further access to detyrosinating *a*-tubulin. Importantly, MARK4 deficiency significantly reduces the loss of ejection fraction following acute myocardial infarction in mouse models (Yu et al., 2021). In addition, enzymatic interfereance by blocking VASH1 or activating TTL effectively decreases stiffness and increases relaxation speed in failing cardiomyocytes of heart failure patients without markedly affecting the intracellular calcium transient in the failing cardiomyocytes of heart failure patients (Chen et al., 2020). As impaired relaxation is a prominent and intractable clinical feature of patients with heart failure with preserved ejection fraction, the findings further support the development of detyrosination as a therapeutic target for diastolic dysfunction.

Taken together, we may conclude that detyrosination PTMs regulate mechanotransduction and contraction among cardiac myocytes, and inhibiting detyrosination could be an appealing and novel therapeutic strategy for improving contractility in the failing heart. Remarkably, the parthenolide treatment, which decreases the fraction of detyrosinated *a*-tubulin, has moved forward to phase I trials for cancer therapies (Yingxian Chen et al., 2018). Therefore, clinical evidence from these kinds of medicines targeting this PTM might be of promising utility in guiding therapy translation to cardiac muscle disorders in the future.

### Tubulin Acetylation

While the acetylation site on tubulin’s lysine 40 (K40) is the most well-characterized, it has remained the most mysterious PTM of tubulin for many years due to its location within the microtubule lumen (L'Hernault and Rosenbaum, 1985; Janke et al., 2005). Unlike other tubulin modifications at C-terminal sites, the most common acetylated PTM refers to the acetyl modified group at K40 of the *a*-tubulin N-terminus (L'Hernault and Rosenbaum, 1985). Acetylation of K40 is majorly mediated by the *a*-tubulin acetyltransferase 1 (ATAT1) (Akella et al., 2010; Shida et al., 2010), while deacetylation is catalyzed by histone deacetylase 6 (HDAC6) (Palazzo et al., 2003) or sirtuin 2 (SIRT2) (North et al., 2003). Since this PTM occurs within the lumen, the enzyme need to enter the lumen to finish its modifying task (North et al., 2003). In addition, other acetylation sites have been proposed by recent proteomic advances (Choudhary et al., 2009; Liu et al., 2015); nevertheless, their distribution and functions have yet to be determined. San acetyltransferase catalyzes another acetylated modification at lysine 252 (K252) site of *ß*-tubulin, which is thought to control microtubule polymerization (Chu et al., 2011). Neverthless, K40 acetylation is more prominent. In terms of primary cilia, K40 acetylation marks long-lived stabilized microtubules in ciliary axoneme and the basal body (Piperno and Fuller, 1985). Earlier research established that acetylation tubulin is a protein abundant throughout the cilium axoneme and is needed for the proper assembly and function of the organelle (Shida et al., 2010). HDAC6 and SIRT2 deacetylases are found to promote primary cilia disassembly in most mammalian cells. Indeed, HDAC6 overexpression leads to the loss of the primary cilium in cancer cells where *a*-tubulin is deacetylation (Michaud and Yoder, 2006). In contrast, the knockdown of HDAC6 can lead to more or longer cilia (Wloga et al., 2017). In the heart, minimal acetylation was identified in adult cardiomyocytes and the ventricle (Belmadani et al., 2004; Kerr et al., 2015), whereas there was a substrate for HDAC6 in ventricular protein (Geeraert et al., 2010; McLendon et al., 2014). Intriguingly, a recent investigation showed the existence of acetylated tubulin on primary cilia exclusively in cardiac fibroblasts (Villalobos et al., 2019).

In general, K40 acetylation has been generally considered to occur on stable microtubule assemblies (Palazzo et al., 2003). However, it remains unknown whether it is directly involved in microtubule assembly. Interestingly, K252 acetylation is one of the few tubulin PTMs that may directly modulate microtubule dynamics, whereas other tubulin PTMs normally do so indirectly *via* altering MAPs. Microtubules tend to depolymerize when the *ß*-tubulin acetylation precludes the incorporation of tubulin dimers (Chu et al., 2011). Furthermore, K40 acetylation can change microtubule mechanical properties and protect them against mechanical breakage (Xu et al., 2017). The loss of K40 acetylation weakens interprotofilament interactions, the loss of which facilitates protofilament sliding and enhances microtubule flexibility. As a result, microtubules tend to be more resistant to mechanical breakage and protected from damage caused by repeated bending (Portran et al., 2017; Xu et al., 2017). Finally, accumulating data shows that acetylated tubulin may further prepare the cell for enhanced autophagic breakdown by augmenting autophagic cargo assembly along microtubules (Geeraert et al., 2010). In light of this, tubulin hyperacetylation has been demonstrated to ameliorate cardiac proteotoxicity by increasing autophagy (McLendon et al., 2014).

Although the specific role of tubulin acetylation has yet to be understood and unraveled, several studies have indicated its link to cardiovascular disorders. Hyperacetylated *a*-tubulin occurs during several forms of cardiomyopathy (McLendon et al., 2014). The modulation functions as an adaptive mechanism in a mouse model of desmin-related cardiomyopathy in which cardiac proteotoxicity aggregates (McLendon et al., 2014). HDAC6 overexpression, which deacetylates *a*-tubulin, increases the aggregation formation. In a mouse model of proteinopathy-induced heart failure, inhibiting tubulin deacetylation by HDAC6 has been shown to be protective where cardiac autophagic flux is enhanced (McLendon et al., 2014). Significantly, inhibiting tubulin deacetylation using suberoylanilide hydroxamic acid (SAHA), a HDAC inhibitor approved by the FDA, can reduce protein aggregate levels and improve cardiac function (McLendon et al., 2014). Similarly, upregulation of acetylated-tubulin, accompanied by deacetylase SIRT2 downregulation and improved cardiomyocyte microtubule stability, is suggested to be a feature of streptozotocin-induced diabetic cardiomyopathy (Howarth et al., 2002).

In cardiac fibrosis and human atrial fibrillation samples, however, acetylated tubulin levels are decreased (Kaul et al., 2014; Tao et al., 2016). The deacetylase HDAC6 lowered the acetylation level of *a*-tubulin in ISO-induced cardiac fibrosis mice. Suppression of HDAC6 reversed *a*-tubulin acetylation and ameliorated cardiac fibroblast proliferation (Tao et al., 2016). Moreover, a recent study confirmed the presence of acetylated tubulin in the primary cilia of cardiac fibroblasts, but the molecular mechanisms are not completely understood (Villalobos et al., 2019). These findings hint at a dynamic and molecular connection between tubulin acetylation and microtubule stability, flexibility and autophagy. While the precise role of acetylation in cardiovascular dysfunction is not entirely known, evidence suggests that HDAC6 may be a feasible therapeutic target (Kaul et al., 2014; Tao et al., 2016).

### Tubulin Glutamylation

Glutamylation is an evolutionarily conserved and functionally significant PTM that is abundantly present on long-lived microtubules such as cilia, neuron axons, mitotic spindles, as well as centrioles (Edde et al., 1990). Tubulin glutamylation patterns and levels catalyzed by different modifying enzymes in distinct organelles, where a single molecule or lateral glutamate peptides of varying lengths at different residues may be added to the either *a*- or *ß*-tubulin C-terminal tails by tubulin tyrosine ligase-like (TTLL) protein family members (Janke et al., 2005). Variation in patterns contributes to microtubule structural and functional heterogeneity in distinct organelles (Verhey and Gaertig, 2007). For example, cilia transition zone microtubules are monoglutamylated, whereas those in the cytoplasm are not glutamylated (Bobinnec et al., 1998), and axonemal or neuronal microtubules are polyglutamylated (Edde et al., 1990; Bobinnec et al., 1998; Pathak et al., 2014). In terms of biochemistry, glutamylation is a reversible process in which side chain glutamates can be removed by cytoplasmic carboxypeptidase(CCP) protein members (Rogowski et al., 2009). The diversity of TPM-generated microtubule subtypes is thought to be preserved by the balanced activity of TTLL glutamylases and CCP deglutamylases, but the functional repertoire remains unclear.

Tubulin polyglutamylation regulates microtubule disassembly in a biphasic manner by promoting enzymatic severance from spastin or katanin (Lacroix et al., 2010; Valenstein and Roll-Mecak, 2016). Additionally, changing degrees and patterns of tubulin polyglutamylation can also fine-tune interactions and thereby affect MAP behavior. Spastin activity is upregulated by early polyglutamylation, while further modification inhibits spastin action (Lacroix et al., 2010). On the other hand, glutamate chains of different lengths had no effect on dynein’s motility or kinesin-13’s depolymerizing activity (Sirajuddin et al., 2014). These findings have far-reaching functional ramifications, as we may hypothesize on the probable MAP controlled by tubulin polyglutamylation based on modification amounts detected in certain cells.

As the glutamylated tubulin accumulates at axoneme structures of the cilia, increased functional evidence thus far hint to essential involvement of glutamylation modification in ciliary structural maintenance and functions (Wloga et al., 2017). Ultrastructurally, polyglutamylation, mostly present on axonemal B tubules (Lechtreck and Geimer, 2000; Orbach and Howard, 2019), directly controls dynein activity and ciliary beating (Kubo et al., 2010; Suryavanshi et al., 2010). For primary cilia, polyglutamylation accumulates at the proximal end of the cilia and indicates its necessity for the correct assembly and function process(Lee et al., 2012; Hong et al., 2018). A recent study in mammalian primary cilia suggests that axoneme polyglutamylation likely anchors polycystins to the cilium’s surface, which is corroborated by the evidence that hypoglutamylation affects the ciliary localization of polycystin 2 (He et al., 2018). Finally, the high degree of polyglutamylation in the centriole’s C tubules of centriole is required for the centrosome to remain intact throughout mitosis (Bobinnec et al., 1998; Gambarotto et al., 2019).

In the heart, ciliated cells were discovered exclusively in fibroblasts of embryonic, neonatal, young and young rat hearts (Kaur et al., 2018), as well as in endothelial cells of cardiovascular arteries (Villalobos et al., 2019). Notably, a recent study suggested that CEP41-mediated ciliary tubulin glutamylation induces endothelial cell migration *via* the HIF1A-Aurka-VEGF pathway, which may be associated with some cardiovascular pathologic processes (Ki et al., 2020). A regulatory network analysis of mRNA-SNP-miRNA sequences revealed that the CEP4-mediated glutamylation at ciliary tubulin stimulated angiogenesis and was involved in cardiovascular disease among type-II diabetes patients (Fan et al., 2021), which has provided new evidence for glutamylation’s role in cardiovascular etiology. However, there is presently no direct evidence supporting the role of microtubule glutamylation in the cardiovascular system. Although glutamylation of tubulin was found in adult cardiomyocytes (Kerr et al., 2015), no evidence linking glutamylation to cardiovascular disease has been found.

### Tubulin Glycylation

While glutamylation is a prevalent microtubule alteration, glycylation has been shown to be specific to mammalian cilia and flagella axonemes (Iftode et al., 2000; Gadadhar et al., 2017a; Gadadhar et al., 2017b). Similar to glutamylation, it is also enzymatically catalyzed by the tubulin tyrosine ligase-like (TTLL) family. (Poly)glycylation PTMs are present as branching peptide chains formed by C-terminal glycine residue(s) (Edde et al., 1990; Bré et al., 1996). This non-binary complex signal is initiated when glycine links to the γ-carboxyl group of a glutamate residue site. In comparison to nine glutamylating enzymes, there are only three glycylation-modifying enzymes expressed in mammalian cells (Rocha et al., 2014). Subsequently, tubulin glutamylation is found in nearly all ciliated organisms (Janke et al., 2005), whereas the occurrence of glycylation rather restricted (Bré et al., 1996). Generally, in mammalian cells, TTLL3 and TTLL8 glycylases are the initiating linking enzymes, while TTLL10 enzymes prolong the chains at the branching point to make polyglycylation (Rogowski et al., 2009). Strikingly, polyglycylase TTLL10 is inactive in humans, which may explain why the side chain is only monoglycylated (Rogowski et al., 2009).

Unlike other tubulin modifications, less is understood about the role of glutamylation modifications in subcellular microtubule structure. In mammals, tubulin glycylation is involved in axoneme stabilization and primary cilia length regulation (Bosch Grau et al., 2013; Pathak et al., 2014; Gadadhar et al., 2017b; Wloga et al., 2017). Monoglycylation is essential for the homeostasis of both motile and primary cilia (Bosch Grau et al., 2013). In terms of polyglycylation, in the absence of TTLL3 and TTLL8, motile cilia completely disintegrate (Bosch Grau et al., 2013) whilst primary cilia are just reduced in quantity, as demonstrated in corneal endothelial cells (CECs) and mouse embryonic fibroblasts (MEFs) (Rocha et al., 2014). In the setting of its cilia-selectivity and in the light of the above findings from different cells, glycylation is expected to be a distinct risk factor for ciliopathies. Although recent studies have reported on the strong link between cilia biology and cardiovascular disorders (Djenoune et al., 2021), whether ciliary glycylations are present in the cardiovascular system and functionally involved in pathophysiology remains uncertain.

## Conclusion and Perspective

Recent functional studies hint at potential implications of microtubules in cardiovascular pathologies. Given the limitations of therapeutic approaches targeting the overall microtubule cytoskeleton, such as colchicine treatment (Stamp, 2014), strategies targeting PTMs allow for effective alternations while modestly altering the overall microtubule cytoskeleton, which largely reduces off-target effects. Thus, PTMs may represent promissing therapeutic targets for increasing contractility in the failing heart. In recent years, insights into the cardiac microtubule network have been focused on the cardiocytes and cilia organelles. In this review, we summarized the current understanding of how cardiac tubulin PTMs control microtubule mechanical properties, dynamics, microtubule-MAP interactions and physiological roles, and their functional implications for the cardiac muscle and cilia signaling. Based on these links, we discussed how altered PTMs may lead to the development of cardiac dysfunction.

In the cardiovascular system, selective microtubule modifications are potentially critical mechanisms, regulating sarcomere anchoring, cardiac mechanotransduction, contractile resistance, autophagic cargo assembly, cilia functioning and angiogenesis, being implicated in the pathogenesis of a range of cardiovascular dysfunctions that may progress to heart failure. Most significantly, the inhibition of detyrosination improved relaxation dynamics considerably in diastolic impaired cardiomyocytes (Yingxian Chen et al., 2018), which raises the prospect that detyrosination-based intervention might be the strategy for treating heart failure patients with preserved ejection fraction, a developing population with no effective treatment options at present. Therefore, deciphering the PTM code will bring fundamentally new insights into the regulative mechanisms for cardiac functions. More importantly, interfering with PTM may represent an attractive and potent therapeutic strategy. Although the influence of tubulin PTMs on microtubule functions is just now being recognized, recent progress is encouraging and exciting new discoveries between PTMs and cardiovascular dysfunctions are expected in the near future.
